# Regulation of inflammatory diseases via the control of mRNA decay

**DOI:** 10.1186/s41232-024-00326-5

**Published:** 2024-03-15

**Authors:** Masanori Yoshinaga, Osamu Takeuchi

**Affiliations:** Department of Medical Chemistry, Graduate School of Medicine, Kyoto University, Kyoto, 606-8501 Japan

**Keywords:** Post-transcriptional regulation, mRNA decay, Autoimmune diseases, mRNA methylation, RNA-binding proteins, Regnase family, Therapeutic intervention

## Abstract

Inflammation orchestrates a finely balanced process crucial for microorganism elimination and tissue injury protection. A multitude of immune and non-immune cells, alongside various proinflammatory cytokines and chemokines, collectively regulate this response. Central to this regulation is post-transcriptional control, governing gene expression at the mRNA level. RNA-binding proteins such as tristetraprolin, Roquin, and the Regnase family, along with RNA modifications, intricately dictate the mRNA decay of pivotal mediators and regulators in the inflammatory response. Dysregulated activity of these factors has been implicated in numerous human inflammatory diseases, underscoring the significance of post-transcriptional regulation. The increasing focus on targeting these mechanisms presents a promising therapeutic strategy for inflammatory and autoimmune diseases. This review offers an extensive overview of post-transcriptional regulation mechanisms during inflammatory responses, delving into recent advancements, their implications in human diseases, and the strides made in therapeutic exploitation.

## Background

The immune system employs a complex biological response known as inflammation to protect the body against harmful stimuli, infections, and tissue injuries [1, 2]. This intricate process involves various cellular and molecular components and their interplay. At the forefront, the innate immune system serves as the mechanism to rapidly respond to the stimulation including microorganisms. Comprising multiple types of cells such as macrophages, neutrophils, and dendritic cells (DCs), the innate immune system acts immediately to recognize foreign pathogens utilizing their innate immune receptors such as Toll-like receptors (TLRs), RIG-I-like receptors (RLRs), NOD-like receptors (NLRs), and cyclic GMP-AMP synthase (cGAS) [3–5]. Upon the engagement of these receptors and their cognate ligands, innate immune cells activate the downstream signaling pathways to induce the nuclear translocation of the key transcription factors such as nuclear factor-kappa B (NF-κB) and interferon response factors (IRFs). These transcription factors promote the production of type I interferons (IFNs) and proinflammatory cytokines including interleukin (IL)-6 and tumor necrosis factor (TNF) and trigger a series of events essential for the containment and resolution of threats [6]. Moreover, innate immune cells present the antigens from pathogens on the cell surface in a complex with the major histocompatibility complex (MHC). This complex is recognized by the CD4^+^ and CD8^+^ T cells via their T cell receptor (TCR), initiating the adaptive immune response coordinated by T and B lymphocytes [7, 8]. CD4^+^ helper T cells promote the activation of naïve B cells, promoting affinity maturation and the production of antibodies highly specific to the pathogens. Also, activated CD8^+^ cytotoxic T lymphocytes eliminate the infected cells by microorganisms. Through the production of molecules such as antibodies and cytokines, innate and adaptive immune cells orchestrate an organismal response to neutralize pathogens.

In addition to the canonical immune cells, non-immune cells have emerged as indispensable contributors to the inflammatory milieu [9]. Endothelial cells, fibroblasts, and epithelial cells are now recognized as active participants in shaping and regulating inflammation [10–12]. Together with immune cells, non-immune cells in various organs including the skin, brain, and intestine govern a diverse range of tissue-specific inflammatory responses. The orchestration maintains tissue homeostasis and defend against exogenous insults.

While the orchestrated collaboration between immune and non-immune cells is vital for mounting an effective inflammatory response in the human body, disruptions in the delicate equilibrium between pro-inflammatory and anti-inflammatory signals can lead to excessive, prolonged, or misdirected immune reactions. These imbalances often result in tissue damage and various pathological conditions, including autoimmune disorders like rheumatoid arthritis and severe inflammatory diseases such as COVID-19-induced acute respiratory distress syndrome (ARDS) [13, 14]. These instances underscore how dysregulated inflammation contributes to both chronic inflammatory conditions and severe infections, highlighting the critical need to comprehend the molecular underpinnings of these diseases for targeted therapeutic interventions.

Post-transcriptional regulation stands as a critical checkpoint in controlling the amplitude and duration of inflammatory responses [14–17]. It plays a pivotal role in mitigating unwarranted or excessive inflammation by regulating the response of immune and non-immune cells. Post-transcriptional mechanisms, including RNA stability, alternative splicing, microRNA-mediated regulation, and RNA modifications, intricately modulate the expression and activity of key inflammatory mediators [18–20]. By exerting precise control over mRNA stability and translation, these mechanisms enable the immune system to dynamically adjust its response to stimulation. Dysregulation in these post-transcriptional processes can tip the balance, leading to sustained or hyperactive inflammatory states characteristic of chronic inflammatory diseases. Moreover, the dysregulation of the post-transcriptional mechanisms has been implicated in the failure and/or alteration in immune cell differentiation. Harnessing the complex regulatory networks that govern post-transcriptional control represents a promising avenue for therapeutic interventions aimed at recalibrating aberrant inflammatory responses, potentially offering more precise and targeted approaches to the management of inflammatory diseases.

In this review, we explore the important role of post-transcriptional regulation in refining inflammatory responses. We discuss the emerging roles of RNA-binding proteins in orchestrating post-transcriptional events and highlight their potential as therapeutic targets for addressing inflammatory disorders. We mainly focused on the recent findings about the roles of RNA-binding proteins and RNA methylations that control mRNA decay in the regulation of inflammation. For the review of other post-transcriptional processes such as microRNA-mediated regulation and alternative splicing, readers are directed to other works in the field [21, 22].

## Overview of mRNA decay machinery regulating immune responses

**Fig. 1:**
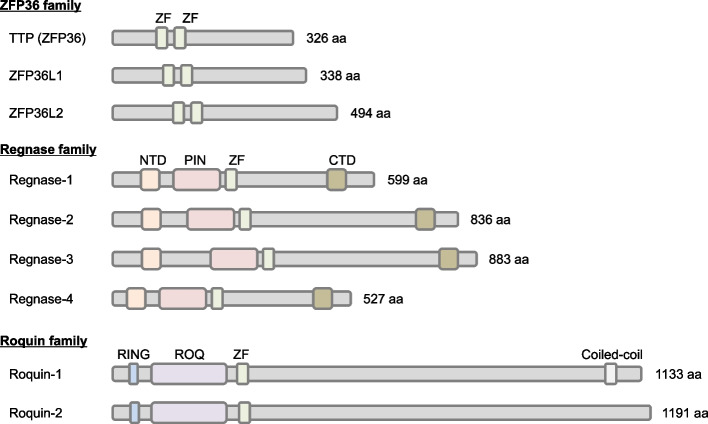
Domain structures of ZFP36, Regnase, and Roquin family proteins. Immune-related RNA-binding proteins (RBPs) such as ZFP36, Regnase, and Roquin family proteins share the common CCCH-type zinc finger (ZF) domain. The TTP family contains tandem ZF domains. The Regnase family features a PIN-like RNase domain, alongside ZF domains, as well as N-terminal (NTD) and C-terminal (CTD) domains. Roquin family proteins possess the ROQ domain, a RING finger domain, and ZF domains. The multiple RNA-binding domains of these proteins facilitate complex RNA recognition

The orchestration of inflammatory responses hinges significantly on the regulation of mRNA decay [14]. Central to this regulation is the inherent instability of mRNAs encoding inflammation-associated genes, notably cytokines and chemokines [23]. Their rapid turnover is largely dictated by specific sequences within their 3′ untranslated regions (UTRs), which serve as recognition sites for a spectrum of RNA-binding proteins (RBPs). These RBPs include ZFP36 family, Roquin-1/2, and Regnase family proteins, classes of proteins that harbor the CCCH-type zinc finger domain (Fig. 1, discussed below). They trigger exo- or endo-nucleolytic cleavage of the mRNAs, exerting precise control over the synthesis of proinflammatory mediators and crucial molecules pivotal in directing immune responses [14].

### Multiple mechanisms to control mRNA turnover in eukaryotes

**Fig. 2:**
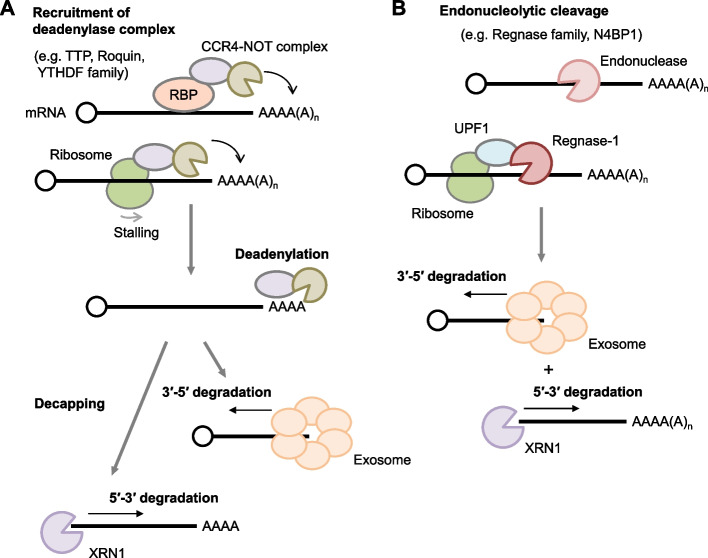
Diverse pathways governing mRNA decay. **A** Various RNA binding proteins (RBPs) like TTP, Roquin, and the YTHDF family, along with stalled translating ribosomes, initiate mRNA decay pathways by recruiting the deadenylase complex. This results in poly(A) tail shortening, enabling access for the cytoplasmic RNA exosome complex to undertake 3′-5′ exonucleolytic decay. Additionally, decapping exposes 5′ ends, targeted by XRN1 for 5′-3′ exonucleolytic decay. **B** An alternative pathway, endonucleolytic mRNA decay, is initiated by endonucleases such as the Regnase family. Regnase-1-mediated mRNA decay requires active translation and the RNA helicase UPF1

The complex interplay between RBPs and mRNA decay machinery underlies mRNA metabolism (Fig. 2). Shielded by post-transcriptional modifications such as 5′ capping and poly(A) tail formation, mature mRNAs are guarded against degradation. These protective moieties cloak the vulnerable 5′ and 3′ bare ends, shielding them from rapid decay. However, once exposed through various mechanisms, the mRNAs begin to undergo degradation.

Among the pathways orchestrating this degradation is the initiation by mRNA deadenylation—a truncation of the poly(A) tail. This process primarily involves the PAN2-PAN3 and CCR4-NOT complexes [24]. As the poly(A) tails diminish, the cytoplasmic RNA exosome gains access to the mRNA's 3′ ends, commencing 3′-5′ exonucleolytic decay. Additionally, decapping mediated by the DCP1 and DCP2 complex, either before or after deadenylation, marks the start of mRNA decay [25]. The exposed 5′ ends are then recognized and cleaved by the 5′-3′ exonuclease XRN1, promoting further degradation. RNA-binding proteins, such as tristetraprolin (TTP, ZFP36) and Roquin-1/2 (RC3H1/2), play pivotal roles in modulating mRNA turnover by controlling the recruitment of deadenylation and/or decapping machinery. Moreover, RNA m^6^A methylation primarily interacts with YTHDF family proteins, which recruits the CCR4-NOT complex and promotes the deadenylation [26, 27].

Alternatively, mRNA turnover can also be kickstarted by direct endonucleolytic decay, exemplified by the Regnase family [28]. Endoribonucleases cleave mRNAs internally, yielding unprotected 5′ and 3′ ends that become targets for XRN1 and the cytoplasmic RNA exosome, respectively. This alternative pathway expedites the degradation of immune-related mRNAs.

Recent findings have highlighted the role of translating ribosomes in recruiting mRNA decay machinery. For instance, Regnase-1-mediated mRNA decay requires mRNA translation [29, 30]. Several other RNA decay mechanisms, such as nonsense-mediated mRNA decay (NMD), no-go decay, and codon optimality-mediated mRNA decay (COMD), rely on translating ribosomes for mRNA degradation [31–34]. While it is unclear how these processes tie into inflammatory responses, investigating their roles in inflammation regulation poses an intriguing avenue for future exploration.

### mRNA marks recognized by RBPs dictate mRNA decay

**Fig. 3:**
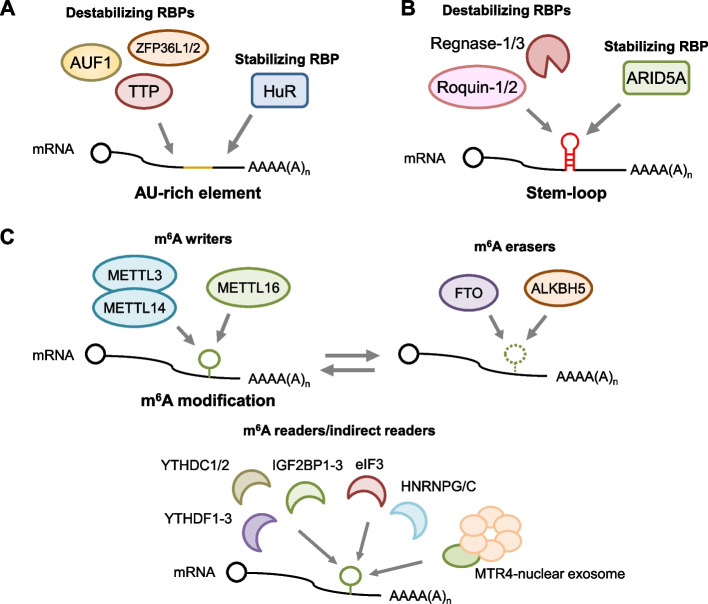
Post-transcriptional mRNA mark recognition by RNA-binding proteins. mRNAs encoding inflammation-related genes contain cis-elements and RNA modifications crucial for post-transcriptional regulation. AU-rich elements (AREs) and stem-loops (**A**, **B**) are cis-elements recognized by multiple RBPs, either destabilizing or stabilizing target mRNAs. Additionally, RNA m^6^A modifications (m^6^A) deposited by m^6^A writer proteins are recognized by direct and/or indirect m^6^A reader proteins (**C**), influencing post-transcriptional regulations. The m^6^A modification can also be removed by m^6^A erasers

The instability of mRNAs encoding inflammation-related molecules primarily stems from inherent sequences within their 3′ UTRs [14]. Specifically, two classes of motifs, termed cis-elements, have been identified—AU-rich elements (AREs) and stem-loops (Fig. 3A, B). AREs represent sequences enriched in A and U, often featuring repetitions of AUUUA pentamers. They are prevalent in 5–22% of human mRNAs, particularly in those encoding proinflammatory genes such as TNF, IL-6, IL-2, and cyclooxygenase 2 (COX2) [35, 36]. AREs are recognized by ZFP36 family members consisted of TTP and ZFP36L1/2 [37] (Figs. 1 and 3A). Also, several ARE-binding proteins including AUF1 (ARE/poly-(U) binding degradation factor 1, also known as HNRNPD) and human antigen R (HuR, also known as ELAVL1), recognize AREs. TTP is one of the most-studied ARE-binding proteins and has a prominent role in the regulation of immune response [38]. TTP harbors tandem CCCH-type zinc finger domains that enable the binding to AREs. Moreover, other members of this family ZFP36L1 and ZFP36L2 also harbors tandem CCCH-type zinc finger domains and are involved in the turnover of ARE-containing mRNAs. In addition to the ZFP36 family members, AUF1 is another RBP with two RNA recognition motifs (RRM) that bind to and destabilize ARE-containing mRNAs [39]. Conversely, HuR recognizes and stabilizes ARE-containing mRNAs [40, 41].

Stem-loop elements, characterized by a pyrimidine–purine–pyrimidine loop sequence, serve as cis-elements primarily recognized by Regnase family proteins and Roquin-1/2 [29, 42, 43] (Figs. 1 and 3B). The Regnase family consists of Regnase-1–4 (ZC3H12A-D, MCPIP1-4), and Regnase-1/3 has been shown to interact with stem-loop elements [29, 43], although the contributions of Regnase-2/4 remains relatively unclear. Their recognition relies on both the structural features and the presence of canonical loop sequences [29, 44]. These elements are abundant within the 3′ UTRs of mRNAs encoding inflammatory mediators and immune-related transcription factors, notably IL-6, IL-2, TNF, inducible T cell costimulator (ICOS), NF-κB inhibitor zeta (NFKBIZ), and NF-κB inhibitor delta (NFKBID) [14]. While both Regnase and Roquin families target similar motifs, their domain structures and mechanisms for mRNA degradation are strikingly different (Fig. 1). Members of the Regnase family harbor a CCCH-type zinc finger domain and a PIN-like RNase domain, whereas Roquin-1/2 are characterized by a CCCH-type zinc finger domain coupled with a unique ROQ domain specific to this family [45]. Regnase-1 employs its endonuclease activity to cleave target mRNAs containing stem-loop structures in a manner dependent on translation and the RNA helicase UPF1, while Roquin predominantly recruits the CCR4-NOT complex to promote the decay of translationally-inactive mRNAs [29, 30, 42, 46]. Therefore, these proteins nonredundantly degrade target mRNAs [29, 47, 48]. There is also evidence suggesting that Roquin collaborates with Regnase-1, forming a complex to facilitate the endonucleolytic decay of target mRNAs [49]. However, further investigation is warranted to elucidate the precise contribution of this mRNA degradation pathway during immune responses. In addition, contrary to the Regnase-1 and Roquin, ARID5A is known to recognize stem-loop elements and counteract with Regnase-1-mediated mRNA decay [50].

RNA methylation serves as an additional layer of post-transcriptional modification influencing mRNA turnover. Among the diverse modifications, *N*^6^-methyladenosine (m^6^A) methylation stands out as one of the most prevalent internal RNA alterations [51, 52]. The installation of m^6^A modifications on mRNAs primarily occurs co-transcriptionally through the action of a canonical m^6^A “writer” complex, constituted by METTL3, METTL14, and other accessory components such as WTAP and ZC3H13 (Fig. 3C). These m^6^A modifications deposited by the METTL3/14 complex are recognized by “reader” proteins such as members of the YTHDF and YTHDC families [53]. Additionally, several direct and indirect reader proteins such as HNRNPG and IGF2BPs are involved in detecting m^6^A modifications [54, 55]. These reader proteins instigate various post-transcriptional regulatory changes, including alterations in mRNA turnover, translation status, and alternative splicing [26, 56, 57]. Moreover, m^6^A modification can be removed by “eraser” proteins including ALKBH5 and FTO [52].

Recent studies have unveiled additional RNA m^6^A methyltransferases, notably METTL16 and ZCCHC4 [58]. These writers selectively deposit m^6^A modifications on a restricted subset of RNA species. METTL16, recognized as a well-conserved m^6^A writer, targets U6 snRNA alongside mRNAs encoding S-adenosyl methionine (SAM) synthases and DNA-repair-related molecules [58–62]. It has been proposed that METTL16 controls SAM concentration by regulating mRNAs encoding SAM synthases, which is crucial for various methylation processes, including METTL3-mediated m^6^A methylation [58, 63]. This suggests a potential indirect regulatory role of METTL16 on the inflammatory response through its impact on METTL3/14 methylation activity. However, recent findings hint at the possibility of METTL16 acting independently of METTL3/14, applying m^6^A modifications to a distinct set of mRNA targets [62]. Interestingly, unlike canonical m^6^A writer complex, METTL16 requires the MTR4-nuclear RNA exosome complex for the regulation of METTL16 substrate mRNAs. Further exploration is important to elucidate their specific roles in modulating the immune response.

## Post-transcriptional control of immune response

Post-transcriptional control orchestrated by RBPs leads to a diverse array of regulations spanning multiple layers of the immune response. These include the regulation of both immune and non-immune cells, along with intricate control over immune cell differentiation and activation. Additionally, self-RNAs necessitate post-transcriptional modifications to evade undesirable immune reactions. This section aims to discuss each of these layers in detail.

### Regulation of innate immune response and signaling

In response to infection by microorganisms, innate immune cells initiate proinflammatory cytokine production such as TNF, IL-6, and type I IFNs [3]. The innate immune stimulation such as the treatment with TLR ligands leads to the transient degradation and/or inactivation of RBPs such as Regnase-1 and TTP, enabling the robust expression of the proinflammatory cytokines at the onset of inflammation [44, 64, 65]. Simultaneously, myeloid cells transactivates the mRNAs encoding a number of RBPs such as Regnase-1/3 and TTP [66], thereby dampening the expression of proinflammatory cytokine mRNAs at the later phase of inflammation. Thus, post-transcriptional regulation emerges as a pivotal factor in modulating the expression of proinflammatory cytokines. Additionally, post-transcriptional mechanisms intricately modulate the response to innate immune signaling and/or the polarization in myeloid cells and other cell types. Upon the proinflammatory stimulation, macrophages undergo the polarization into the classically activated phenotype with proinflammatory properties [67]. During this polarization, METTL3 has been shown to be upregulated, which further promotes the proinflammatory phenotype [68]. These lines of evidence suggest that the immune-related RBPs are integrated into the regulation of the activation of innate immune system. The specific roles of RBPs in the regulation of the innate immune system are discussed below.

A large body of studies using the RBP-deficient mice have shown that the regulation of proinflammatory cytokine mRNAs involves multiple RBPs. For instance, TTP facilitates the decay of several ARE-containing mRNAs, including *TNF, CSF2*, *IL6*, and *IL5* mRNA, suppressing the development of autoimmune-like symptoms like arthritis and nephritis [69–72]. Roquin and Regnase-1 also contribute significantly to the degradation of mRNAs encoding various proinflammatory mediators including TNF, IL-6 and chemokines [29, 42, 66]. Conversely, ARID5A has been shown to counteract *IL6* mRNA degradation by Regnase-1, whose depletion leads to protection against LPS-induced septic shock in vivo [50]. In addition to the RBPs that binds to AREs and stem-loops, studies indicate that m^6^A modifications regulate the production of type I IFNs and interferon-stimulated genes (ISGs) after exposure to double-stranded DNA [73]. This effect involves the direct destabilization of *IFNB* mRNA through YTHDF2 and is counteracted by ALKBH5 [73, 74]. Therefore, post-transcriptional mechanisms coordinate multiple RBPs to fine-tune the expression of proinflammatory cytokines.

The transduction of IFN signaling is subject to intricate post-transcriptional regulation. It has been reported that Regnase-3 plays a crucial role in suppressing IFN signaling in myeloid cells in vivo, although the underlying mechanism is obscure [75]. Also, the m^6^A-independent function of YTHDF3 involves downregulating ISGs by activating the transcriptional corepressor FOXO3 [76]. METTL3-mediated m^6^A modifications on *Stat1* and *Irf1* mRNAs restrain IFN-γ signaling [77]. Additionally, RBPs such as DDX6 and RBM47 regulate type I IFN signaling and/or ISG expression [78, 79]. These findings clearly indicate that the IFN signaling is intricately regulated by a concerted action of multiple RBPs. This tight association between IFN signaling and RBPs underscores the need for comprehensive exploration into the regulatory roles of RBPs in this process in the future.

RBPs play a crucial role in the development and function of innate immune cells. Specifically, m^6^A modification has been found to regulate macrophage activation. Evidence suggests that METTL3-mediated m^6^A modifications promote proinflammatory signaling, including TLR and NF-κB pathways, or facilitate the polarization towards the classically-activated phenotype [68, 80, 81]. However, conflicting reports propose the suppression of macrophage activation or proinflammatory polarization by m^6^A regulators such as METTL3 and YTHDF2 [82, 83]. Consequently, the precise roles of METTL3-mediated m^6^A modifications in macrophages remain a subject of debate, warranting further investigation.

Additionally, m^6^A modification plays a pivotal role in various innate immune cell types. METTL3 has been shown to promote activation and T cell priming in DCs through its catalytic activity [84]. Interestingly, mice with YTHDF1-deficient DCs reportedly exhibit enhanced cross-priming activity and anti-tumor immunity [85], highlighting the reader-specific function of m^6^A modification. The authors found that YTHDF1 binds and promotes the translation of mRNAs that encode lysosomal cathepsins, which act as negative regulators of cross-presentation [85]. In neutrophils, METTL3-mediated m^6^A methylation promotes the TLR signaling activation, thereby controlling the release from the bone marrow into circulation upon LPS stimulation in vivo [86]. In natural killer (NK) cells, METTL3-mediated m^6^A modification, recognized by YTHDF2, is crucial for effector function and survival [87, 88]. Moreover, m^6^A modification is required for the cell types that bridge innate and adaptive immune response such as invariant natural killer T (iNKT) and γδT cells. METTL3-mediated m^6^A modification is essential for iNKT cell development, survival, and functionality [89]. Furthermore, ALKBH5 restricts the development of γδT cells [90]. These findings underscore the importance of m^6^A modification in the development of innate immune cells and the immune response orchestrated by these cells.

### Regulation of adaptive immunity

Once the innate immune cells are activated upon the infection of microorganisms, these cells present antigens from the engulfed microorganisms in a complex with MHC molecules. The complex is recognized by TCR and triggers the activation of downstream TCR signaling in T cells, igniting the effector function of T cells. Simultaneously, TCR signaling has been shown to activate the MALT1 paracaspase to cleave a number of RBPs such as Regnase-1 and Roquin, which promote the degradation of mRNAs important for T cell effector functions [91, 92]. This dynamic dampening of post-transcriptional mechanisms upon TCR stimulation supports the full activation of T cells, thereby governing adaptive immune response. Moreover, antibody production and B cell response is also controlled by multiple RBPs, implying the central role of the post-transcriptional mechanisms in the control of adaptive immune response.

Roquin and Regnase-1 are closely linked with the regulation of the T cell response and therefore, they are one of the most studied molecules in this context. Loss-of-function mutations or deficiencies in Roquin proteins result in aberrant T cell activation and spontaneous accumulation of follicular helper T (Tfh) and Th17 cells in vivo [92–94]. Similarly, Regnase-1 deficiency in T cells triggers spontaneous T cell activation in vivo [91]. This is attributable to the role of Roquin and Regnase-1 in degrading the mRNAs encoding T cell activating factors such as IL-2, ICOS, IL-6, and NFKBIZ [91, 92, 94]. Additionally, deficiency in Regnase-1 has been shown to enhance the anti-tumor immunity of CD8^+^ T cells by improving effector functions due to the upregulation of BATF, a critical Regnase-1 target for this phenotype [95]. Simultaneous loss-of-function mutations in Regnase-1 and Roquin in T cells intensify spontaneous T cell activation and Th1 differentiation, culminating in systemic inflammation and cardiac fibrosis in vivo [47]. As described above, T cell activation appears to dampen the repression by Regnase-1 and Roquin, as these proteins are cleaved by MALT1 paracaspase [91, 92]. Intriguingly, the constitutively active mutant of MALT1 induces spontaneous T cell activation and severe autoimmune inflammation [96]. Recent reports indicate that this phenotype is significantly alleviated by concurrent mutation in the MALT1 cleavage site of Roquin-1 [97]. Furthermore, mice harboring MALT1-insensitive Roquin-1 display resistance to experimental autoimmune encephalitis (EAE). These discoveries highlight the critical role of dynamic regulation of RBP activity in T cells for immune response control.

The canonical components of the m^6^A writer complex play pivotal roles in the proliferation and activation of naïve T cells, while it plays an essential role in  regulatory T cells (Treg) as well [98–100]. This is due to the repression of key negative regulators of IL-7-STAT5 signaling such as SOCS1, SOCS3, and CISH by m^6^A modifications [98]. Also, it has been proposed that the T cell-specific deletion of WTAP leads to augmented TCR signaling and activation-induced cell death [101]. ALKBH5 promotes autoimmune pathology by upregulating proinflammatory mediators such as IFN-γ and CXCL2 [102]. Furthermore, the development of Tfh cells, crucial for germinal center formation, necessitates METTL3 [103]. Therefore, m^6^A modification is an essential post-transcriptional regulator in T cells.

B cell development and activation are also intricately regulated by RBPs. ZFP36L1 and ZFP36L2 play redundant roles in B cell development in the bone marrow by promoting the cell quiescence [104], with ZFP36L1 specifically required for marginal zone B cells and antibody-secreting cells [105, 106]. Similarly, Regnase-1/3 redundantly contribute to early B cell development by degrading *NFKBIZ* mRNA, while deficiencies in Regnase-1, but not Regnase-3, result in aberrantly activated B cell phenotypes [43, 107]. Recent studies suggest that TIA1 and TIAL1 collaboratively regulate the splicing of DNA repair genes, influencing B cell lymphopoiesis [108]. Also, HuR has been shown to be required for B cell proliferation and activation, and germinal center reaction [109, 110]. Moreover, m^6^A deposition plays a vital role in various stages of antibody production. Studies indicate that METTL3-mediated m^6^A deposition is essential for early B cell development and the germinal center reaction [111–113].

### Regulation of non-immune cells

Post-transcriptional mechanisms have a significant influence on the function of non-immune cells that intersect with immune regulation, such as epithelial cells and fibroblasts. Notably, Regnase-1 has been implicated in regulating a variety of non-immune cells, alongside its role in innate and adaptive immune cells.

The intestines serve as a critical site for constant recognition of food antigens and commensal bacteria by the immune system. The epithelial lining, acting as a barrier against unwanted pathogens, also needs to absorb nutrients from ingested food. Regnase-1 is implicated in facilitating dietary iron uptake in the duodenum, a crucial nutrient for both host and pathogens, at the steady state [114]. Additionally, it plays a role in intestinal epithelial regeneration following dextran sodium sulfate (DSS) treatment, a mouse model of colitis [115].

The lung and skin are organs that require effective clearance and control of harmful pathogens. In lung epithelial cells, Regnase-1 downregulation contributes to regulating bacterial infections from the airway [116]. Also, in the skin, Regnase-1 in keratinocytes suppresses IL-17R signaling, thus inhibiting psoriasis development [117].

Adipose tissue hosts numerous immune cells and plays a crucial role in systemic energy metabolism [118]. Regnase-1 in adipocytes has been linked to adipocyte differentiation in vitro [119], although in vivo studies have not fully elucidated its specific role. The multifaceted functions of a single RBP across diverse cell types highlight its potential to modulate tissue-specific immune reactions. Investigating the roles of other RBPs in various contexts could unveil additional layers of complexity and immune regulatory networks.

### Post-transcriptional modification of self-RNA and the control of global RNA decay

**Fig. 4:**
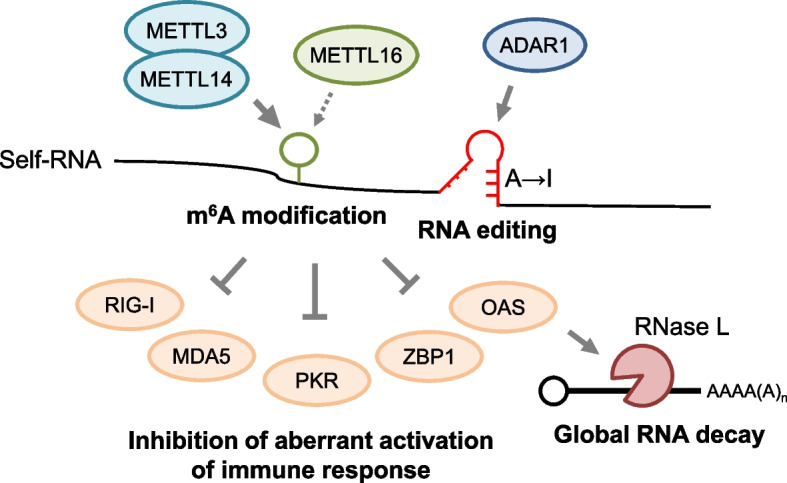
Post-transcriptional control to evade immune responses and global RNA decay. Intracellular recognition of double-stranded RNAs (dsRNAs) activates innate immune signaling, initiating global RNA decay via the OAS-RNase L system. Processes like RNA editing and m^6^A modification play pivotal roles in altering RNA secondary structures, facilitating evasion from innate immune responses against self-RNAs

Cytoplasmic RNAs are under constant scrutiny by the RLRs including MDA5 and RIG-I, which recognizes double-stranded RNAs (dsRNAs) that are not commonly observed in self-RNA [120]. Following sensing of viral dsRNAs, RLRs trigger signaling pathways that result in the production of type I IFNs. Under physiological conditions, RBPs and post-transcriptional modifications keep the burden of dsRNAs low (Fig. 4). However, the failure to suppress the immune response against self RNAs can lead to a range of human diseases known as Aicardi–Goutières syndrome (AGS), characterized by heightened type I IFN production [121]. Moreover, the activation of type I IFN signaling is known to trigger the intracellular RNA decay by the OAS-RNase L system, which serves as a mechanism to cleave viral and cellular RNAs [122]. The recognition of dsRNAs by OAS initiates the production of 2′-5′ oligoadenylate from ATP, which facilitates the formation of active dimerized RNase L. The RNA cleavage by RNase L produces ligands for RIG-I and MDA5, thereby further instigating the inflammatory response [123]. Hence, maintaining tight control over self-RNA modifications is a crucial component in maintaining immune homeostasis.

The action of ADAR1, which mediates A-to-I editing of RNAs, plays a vital role in mitigating intracellular dsRNA levels [124]. This editing process alters the secondary structure of dsRNAs, thereby aiding in evading recognition by intracellular dsRNA sensors such as MDA5, PKR, and ZBP1 [125, 126]. This system is crucial for suppressing the production of type I IFNs and preventing autoimmune-like pathologies triggered by the recognition of self-RNAs. Notably, AGS-causing mutations in the ADAR1 gene found in both human and mouse models, genetically corroborate the importance of this system in preventing autoimmune reactions [127, 128].

RNA m^6^A modification is proposed to play a role in shielding self-RNA from innate immune sensors. Initially, in vitro studies suggest that m^6^A-modified RNAs exhibit reduced immunogenicity compared to unmodified RNAs, evading the activation of TLRs and RLRs [129, 130]. This evasion mechanism might be associated with m^6^A’s ability to regulate RNA secondary structures [131–133]. Inhibiting or eliminating METTL3 increases intracellular dsRNA levels, facilitating recognition by RIG-I or MDA5, subsequently activating type I IFN signaling and RNase L activation [134–136]. Additionally, deletion of the m^6^A writer METTL16 heightens expression of ISGs, although the underlying mechanisms remain largely unclear [62]. Thus, m^6^A modification emerges as a novel element in shielding self-RNA from recognition by the innate immune system.

## Relevance to human inflammatory diseases

RBPs have been strongly linked to the pathogenesis of various human inflammatory diseases, with Regnase-1 particularly implicated in multiple pathological conditions associated with abnormal inflammation. In idiopathic pulmonary fibrosis, group 2 innate lymphoid cells (ILC2), which are the important player in the type 2 immune responses by producing IL-5 and IL-13, have been implicated in the disease pathogenesis [137]. In this context, a negative correlation has been established between Regnase-1 protein expression in ILC2 and their quantity in bronchoalveolar lavage fluid in these patients [138]. Similarly, Regnase-1 expression levels in peripheral blood mononuclear cells (PBMCs) from pulmonary artery hypertension patients have shown an inverse relationship with disease severity [139]. Correlations have also been observed between Regnase-1 expression in PBMCs and the size of neurological lesions in multiple sclerosis patients [140]. Augmented expression of Regnase-1 has been demonstrated in human psoriatic skin lesions [117]. Additionally, gain-of-function mutations in the *ZC3H12A* gene (encoding Regnase-1) have been identified in intestinal epithelial cells of ulcerative colitis patients [141, 142]. These findings collectively underscore the pivotal role of Regnase-1 in the pathogenesis of inflammatory diseases.

In addition to the clinical relevance of Regnase-1, numerous studies have elucidated the connection between other RBP mutations and inflammatory diseases. In a Japanese hereditary antithrombin patient with autoimmune disease-like symptoms, a substantial genomic deletion encompassing the *RC3H1* gene, encoding Roquin-1, has been documented [143]. Additionally, homozygous nonsense mutations in Roquin-1 lead to hyperinflammation in relapsing hemophagocytic lymphohistiocytosis (HLH) [144]. Furthermore, several genome-wide association studies have highlighted an association between Regnase-3 and the development of psoriasis [145, 146]. Notably, disease-associated single nucleotide polymorphisms (SNPs) have been linked to augmented Regnase-3 expression [147]. Moreover, mutations in the *ZFP36* gene (encoding TTP) have been identified in patients with rheumatoid arthritis [148, 149]. Additionally, single nucleotide polymorphisms associated with the *ZFP36L1* gene have been linked to Crohn’s disease and idiopathic juvenile arthritis [150, 151]. These collective findings underscore the pivotal role of RBPs in the pathogenesis of human inflammatory diseases.

## Therapeutic approaches targeting post-transcriptional regulation

Current treatments for inflammatory and autoimmune diseases primarily involve immunosuppressants and monoclonal antibodies targeting proinflammatory cytokines and their receptors [152, 153]. While these therapies are effective for some patients, they are not universally successful and certain individuals may not respond well or may experience adverse effects, including immunocompromisation. Consequently, there is a pressing need to explore novel therapeutic avenues to address these limitations.

Given the fine-tuning role of post-transcriptional regulation in inflammatory responses, the specific intervention in RBP-mediated mechanisms emerges as a promising therapeutic strategy for various autoimmune and inflammatory diseases [154]. Furthermore, in specific scenarios like infections, vaccinations, and anti-tumor immunity, augmenting immune reactions can be advantageous. The intricate modulation of RBPs offers a potential avenue to precisely enhance immune responses in a more targeted and controlled manner. Here, we present several examples that demonstrate therapeutic interventions harnessing post-transcriptional mechanisms to modulate inflammatory responses.

### Antisense oligonucleotide (ASO)

Antisense oligonucleotides (ASOs) are a class of compounds that bind complementarily to specific target sequences [155]. Depending on these sequences, ASOs can have various biological effects, such as translation inhibition, RNA degradation, and exon skipping. For instance, in the treatment of spinal muscular atrophy, an ASO therapeutics aiming to induce exon inclusion in SMN2 transcripts, Nusinersen (Spinraza), has been clinically approved to address neurological symptoms [156]. These advancements mark the dawn of RNA medicine.

Recently, ASOs have been employed to disrupt mRNA secondary structures, thereby interrupting the cis-elements targeted by Regnase-1 or Roquin [140]. Regnase-1 contains its target motifs within its 3′ UTR, creating a self-regulatory negative feedback system [44, 140]. ASOs that disrupt these motifs enhance the expression of Regnase-1, enabling it to suppress proinflammatory cytokines and chemokines. Therapeutically administered ASOs in mice have successfully ameliorated several models of inflammatory and autoimmune diseases, serving as proof-of-concept for mRNA structure-disrupting therapy [140]. Similarly, disruption of stem-loops in *Nfkbiz* mRNA, which are targeted by Regnase-1/3, promotes the myeloid cell production from hematopoietic stem cells at the expense of lymphoid cells [43]. This approach holds promise for targeting various mRNA secondary structures to modulate immune reactions.

### Genetic ablation of RBPs

RBPs function as negative regulators of inflammatory responses, and their removal amplifies inflammatory reactions, presenting potential benefits in anti-tumor immunity. This particularly aids in generating potent chimeric antigen receptor (CAR)-T cells, ex vivo engineered T cells designed to target tumor antigens. Research indicates that the deletion of Regnase-1 enhances CAR-T cell efficacy in tumor clearance by rescuing them from exhaustion and promoting proliferation [157]. Furthermore, a genome-wide CRISPR screen for CD8^+^ T cell fitness revealed that Roquin-1 depletion promotes T cell expansion and anti-tumor immunity by upregulating IRF4 [158]. Concurrent disruption of Regnase-1 and Roquin-1 further heightens anti-tumor immunity [48]. These findings underscore the potential of leveraging the anti-inflammatory roles of RBPs to bolster the immune response against tumors.

Recently, several techniques have been reported to induce the genome editing of immune cells or HSCs in vivo. These include the delivery of adeno-associated virus vectors and lipid nanoparticles (LNPs) [159, 160]. These methodologies can be harnessed to deplete RBPs in vivo, potentially avoiding the need for costly ex vivo generation process of CAR-T cells. This line of research, together with the study of RBP functions, will make the RBP-targeted CAR-T cell therapy a readily available, universally applicable treatment.

### Compounds that inhibit or modulate RBP function

The functions, activity, and subcellular localization of RBPs are tightly regulated by multiple factors, including protein kinases, proteases, and ubiquitin ligases [44, 64, 91, 161–163]. Targeting these regulatory systems could hold therapeutic promise in mitigating inflammatory diseases. For example, the SMG1 inhibition, which disrupts Regnase-1-mediated mRNA decay, has demonstrated potential by promoting the activation and proliferation of DCs in vitro [30]. Such interventions could be harnessed to enhance immune responses for effective vaccination and combating infections.

Additionally, therapeutic targeting of m^6^A modification is under exploration. Various inhibitors for the canonical m^6^A writer complex have been reported and hold potential for therapeutic use [135, 164]. Recent studies suggest that inhibiting METTL3 increases the immunogenicity of cancer cells, thereby enhancing anti-tumor immunity [135]. This avenue might also be viable for modulating inflammatory diseases considering the pivotal role of m^6^A modification in the inflammatory response. However, since METTL3 inhibition affects diverse immune responses and cell differentiations, future studies will need to focus on delivering the inhibitors in a cell type-specific manner to elicit the desired therapeutic effects.

## Future perspectives

Here we have discussed the multifaceted roles of post-transcriptional regulation in inflammatory diseases. Further elucidating the specific contributions of RBPs, especially the TTP, Roquin, and Regnase families, in orchestrating post-transcriptional regulatory mechanisms promises deeper insights into the fine-tuning of immune reactions. Additionally, comprehending the intricate crosstalk between these RBPs and RNA modifications, such as m^6^A methylation, holds the potential to unveil novel mechanisms underlying immune regulation. Moreover, it has been reported that a large number of RBPs are expressed in immune cells, and future studies will uncover a crucial role of these novel RBPs in modulating immune responses [165, 166]. These essential studies will serve as the foundation for unlocking the potential of RNA-based therapeutics and RBP-targeting interventions, paving the way for promising frontiers in managing immune-related disorders.

## Data Availability

Not applicable.
